# Effort-reward imbalance and its association with sociocultural diversity factors at work: findings from a cross-sectional survey among physicians and nurses in Germany

**DOI:** 10.1007/s00420-022-01947-4

**Published:** 2023-01-05

**Authors:** Anna Schneider, Christian Hering, Lisa Peppler, Liane Schenk

**Affiliations:** Institute of Medical Sociology and Rehabilitation Science, Charité–Universitätsmedizin Berlin, Charitéplatz 1, 10117 Berlin, Germany

**Keywords:** Sociocultural diversity, Effort-reward imbalance, Physician, Nurse, Hospital, Quantitative studies

## Abstract

**Objective:**

Due to staff shortages and reports of high work stress, work conditions of hospital physicians and nurses receive wide attention. Additionally, sociocultural diversity of the workforce and patient population is increasing. Our study aim is to analyze how individual and organizational diversity-related factors are associated with the experience of staff’s work stress.

**Methods:**

A cross-sectional online survey was conducted with healthcare staff from 22 acute hospitals operated by two healthcare organizations in Germany in 2018. Sociodemographic, occupational and organizational factors were surveyed. Participants further reported work conditions related to the sociocultural diversity of colleagues and patients. Effort-reward imbalance (ERI) was measured with the German short version. Multivariable regression models were calculated with ER ratio as an outcome.

**Results:**

*N* = 800 healthcare staff were included. Variables associated with higher ERI were longer work experience (*β* = 0.092, *p* < 0.05), not holding a leading position (0.122, < 0.01), being a witness (0.149, < 0.001) or victim (0.099, < 0.05) of discrimination at one’s own ward, reporting frequent burden due to language barriers with patients (0.102, < 0.01) and colleagues (0.127, < 0.001), and having restricted access to translators at work (0.175, < 0.001). Factors associated with lower ERI were having a first generation migration background (− 0.095, < 0.05) and being a physician (− 0.112, < 0.05).

**Conclusions:**

Catering to the needs of healthcare personnel in dealing with the additional effort related to language barriers at work, e.g., readily available translator services, and creating non-discriminatory work environments might be one cornerstone for the prevention of work-related ill health and retention of qualified hospital staff.

**Supplementary Information:**

The online version contains supplementary material available at 10.1007/s00420-022-01947-4.

## Background

Healthcare professionals frequently experience work stress stemming from adverse psychosocial work conditions (Hasselhorn et al. 2004; Le Huu et al. 2022), although considerable variation in prevalence and magnitude of stress levels applies with regard to the country, healthcare setting and profession under study (Nguyen Van et al. 2018). However, little is known about the impact of the requirements placed on healthcare staff stemming from the increasing sociocultural diversity of work teams and patients on individual’s work stress in healthcare. In this study, we understand sociocultural diversity as a multidimensional perspective on the close connection of social and cultural aspects of social groups (Betancourt et al. 2003). These aspects are associated with disparities rooted in social structures and result in differences in values, behaviors, and access to social positions and resources (Hradil 2000). Besides taking into account differences in the sociodemographic composition of healthcare professional groups, we placed our focus on diversity aspects stemming from individuals’ intersections with migration and (foreign) languages at work. The diversity of the general labor force is increasing for decades, e.g., regarding employees’ gender, training, qualification, and age (Siegrist 2016). However, specifically in the case of the German healthcare system, national shortages of physicians and nurses are partly compensated by the recruitment of qualified personnel abroad (Williams et al. 2020), leading to a proceeding diversification in care and medical teams with regard to team members’ sociocultural backgrounds and language skills. Furthermore, the sociocultural diversity of patients increases, also resulting in higher demands for interculturally appropriate provision of healthcare services (Degrie et al. 2017). Increased sociocultural diversity in work teams requires staff flexibility and individual and team resources to adequately deal with resulting differing individual needs, interests and backgrounds and to ensure fair and inclusive workplaces (Pasca and Wagner 2011). Thus, analyzing associations of sociocultural diversity work factors in conjunction with an established work stress model follows up on the demands of rethinking and extending traditional occupational health theories in consideration of social changes (Pasca and Wagner 2011; Polanyi and Tompa 2004).

Various instruments are available for the study of psychosocial work environments with differences in their (lack of) theoretical underpinning, focus on specific work aspects and their evaluation, availability of measurement instruments in different languages, psychometric properties of these instruments and their capability to explain the health of employees (Tabanelli et al. 2008). Due to its strong theoretical foundations, the availability of a valid short measurement instrument in German language and its empirical relevance for the explanation of physical and mental well-being in healthcare professionals, the effort-reward imbalance (ERI) model was chosen to assess work stress in our study. The ERI approach is based on a well-established theoretical model which is widely used in organizational psychology and medical sociology to analyze a variety of different psychosocial work environments and their effects on individual health (Siegrist 1996; van Vegchel et al. 2005). Central to the ERI model is the premise of social reciprocity in the relationship between employee and employer, which is symbolized by the work contract (Siegrist and Li 2020). Thus, employees have certain expectations from their organization, e.g., regarding workload, the cooperation with colleagues and supervisors and appropriate reward in return for their commitment at work. Unmet expectations and a perceived imbalance between effort and reward violates workers’ expectations of the social contract of reciprocity (Bakker et al. 2000). Resulting lower self-esteem, lower sense of control, increased negative feelings and related psychobiological responses measured by “cardiovascular, hormonal, immune and inflammatory markers” negatively affect workers’ physical and mental health, especially if experienced repeatedly (Siegrist 2016; Siegrist and Li 2020). In empirical studies of general populations, the experience of work stress was associated with different mental and physical health disorders (Siegrist and Li 2020; van Vegchel et al. 2005), e.g., coronary heart disease (Backé et al. 2012) and depression (Rugulies et al. 2017). In contrast to other prominent work stress models, such as the Job Demands-Control model (Karasek Jr 1979), the ERI model further introduces the concept of overcommitment, which describes an individual personal characteristic associated with an unfavorable pattern of excessive engagement at work (Siegrist 2016). Affected individuals are especially prone to experience an imbalance due to their exaggerated work effort and high need for approval, which entails that exerted effort often fails to be met with adequate reward in the perception of the employee. Overcommitment is theorized to mediate the association of effort-reward imbalance and health (Siegrist et al. 2004).

Previous empirical research repeatedly showed the detrimental effects of ERI on several health outcomes in health professionals. On the individual level, higher ERI was associated with higher burnout risk (Bakker et al. 2000; Hasselhorn et al. 2004; Häusler et al. 2018), and worse self-rated health in nurses (Ge et al. 2021) and physicians (Buddeberg-Fischer et al. 2008), whereby this relationship was mediated by lower job satisfaction and lower work engagement in nurses (Ge et al. 2021). Regarding work performance, an imbalance between effort and reward was associated with worse (self-perceived) quality of care in German physicians (Klein et al. 2011; Loerbroks et al. 2016) and work ability in a sample of different hospital health workers in Brazil (Martinez et al. 2015). Regarding professional outcomes, higher imbalance ratios of effort and reward were associated with increased thoughts of leaving the current employer or the profession among nursing staff (Derycke et al. 2010; Gräske et al. 2021; Hasselhorn et al. 2004; Li et al. 2013; Schug et al. 2022) and lower work satisfaction in a sample of newly graduated nurses and physicians in Sweden (Enberg et al. 2013). Furthermore, higher ERI was associated with more short-term sickness absences in nurses (Schug et al. 2022).

In comparison to health outcomes, the antecedents and concomitants of ERI in health professionals have been studied more rarely. ERI was associated with different sociodemographic (i.e., age, gender, educational level) and organizational factors (i.e., job position, type of employment, monthly income, job experience, duration of daily break times, work day, frequency of night shifts, working hours per week, adequacy of staffing and the experience of workplace violence) in healthcare professionals (Ohlander et al. 2015; Tian et al. 2021). However, the topic of sociocultural diversity work factors and associations with the experience of ERI in healthcare are understudied up to date (Pasca and Wagner 2011). On one hand, the individual’s belonging to a minority group may have effects on his or her experience and assessment of work factors and resulting occupational well-being. Thus, migrated workers might perceive more effort due to the need to overcome language barriers at work and to prove their abilities in collaboration with new colleagues and supervisors (Pasca and Wagner 2011). However, witnessing or experiencing discrimination at the workplace was associated with lower job satisfaction (Shields and Price 2002), poorer mental health (Hu et al. 2019) and higher stress levels (Boateng and Adams 2016) in healthcare professionals. Discrimination events at work may, thus, violate the social contract between the health professional and his or her employer in a particularly pronounced way by disappointing employee’s trust in reciprocal and fair relationships and negatively impact their self-esteem and emotions (Siegrist 2016), e.g., by evoking feelings of organizational injustice leading to adverse effects on health (Topa et al. 2016). On the other hand, additional challenges in the navigation of an increasingly socioculturally diverse workplace, e.g., language barriers with patients or colleagues or the unavailability of translation services in case of need, might increase work stress of all professionals with already tight schedules and high work demands.

Considering the high prevalence of work stress in healthcare professionals from previous studies and the increasing sociocultural diversity of the work force in Germany, the aim of our study was to analyze how the experience of an imbalance of effort and reward at work was related to individual and organizational characteristics with specific focus on sociocultural diversity work factors. To investigate divergent associative patterns with the single constructs of the ERI model, we further investigated associations between effort, reward, and overcommitment with work factors, separately.

## Methods

### Study population

The study population consisted of physicians and nurses employed in one of 22 acute care hospitals operated by two health organizations in Germany. The standardized online survey was conducted between May and November 2018. The overall aim of the survey was to collect data on the state of the intercultural orientation of both health organizations, the cultural competence of employees and work-related stressors and resources (Schenk et al. 2022). The invitation to take part in the online survey and a respective link to the webpage was sent via email to the institutional addresses of physicians and nurses by the personnel departments of the organizations. Additionally, in organization B, a printed invitation letter with a scannable QR code was sent with the pay slip to employees, since not all employees held institutional email addresses. Two reminders were sent after the first invitation to increase response rates.

A complete survey was pursued with an approximate target population of *n* = 6500 healthcare professionals (*n* = 2500 physicians and *n* = 4000 nurses) in organization A and *n* = 7039 (*n* = 1254 physicians and *n* = 5785 nurses) in organization B. Due to low participation rates of physicians, a paper-and-pencil survey was offered to physicians of two randomly selected hospitals of organization B. The final sample size after data correction amounted to *n* = 800 participants for this analysis (response rate: 5.9%, i.e., data from 800 out of 13,539 invited individuals were used).

## Measures

### General characteristics on the participant and study setting level

The online questionnaire included questions on the following characteristics of participants: gender (male or female), age and job experience (in years), job role (nurse or physician), leading position (yes or no), employment status (temporary or permanent), and work status (full time or part time). Participants further reported their respective affiliation to one of the two study organizations (subsequently anonymized into institution A and institution B for data protection reasons).

### Sociocultural diversity factors on the participant and study setting level

We asked participants about their personal migration experience and the migration experience of their parents, which we subsequently categorized into no migration experience, first generation and second generation migration background as suggested by Schenk et al. (2006). Participants also reported experiences of discrimination as a witness (yes or no) or as a victim (yes or no) at their own wards. As an important factor with regard to culture-related research questions, we applied the short form measure of cultural competence and its subscales measuring participants’ culture-related knowledge, skills, and cognitive orientation (SFCQ; Thomas et al. 2015). With regard to work factors, the survey further inquired participants’ perceived burden at work due to language barriers when interacting with patients as well as the perceived burden due to language barriers when interacting with colleagues and supervisors on a five-point scale from ‘always’ to ‘never’. One further question addressed the respondents’ possibility to consult an interpreter in case of language barriers at work. The former two items were subsequently recoded and all three items were treated as metric Likert-type scales. With regard to organizational features, two separate questions inquired the estimated percentage share of employees with a migration experience and the estimated percentage share of patients with a migration experience on the respondents’ ward.

### Effort-reward imbalance

The validated short version of the ERI questionnaire was used to measure effort with three items, reward with seven items and overcommitment with six items (Siegrist et al. 2009). The short version was applied due to its documented ability to measure crucial aspects of psychosocial work environments relevant to health by reference to the original theoretical construct (Siegrist et al. 2009). Furthermore, the brevity of the instrument in comparison to the original ERI questionnaire was deemed particularly suitable for use in an online survey to limit the time required to complete the survey. Although we did not examine associations with health outcomes of participants, we included overcommitment in our analyses to exploratively assess its connections with sociocultural diversity work factors. In the ERI questionnaire, respondents rate on a 4-point Likert scale to what extent they agree to the respective statement with regard to their actual work situation (Montano et al. 2016). Internal consistency for the three subscales (Cronbach’s α) was satisfactory: effort (*α* = 0.724), reward (*α* = 0.709) and overcommitment (*α* = 0.767). For statistical analyses, the ratio between effort and reward was calculated as a separate variable, by building the ratio between the sum score of effort (E) and the reward sum score (R) corrected by the ratio of items in each subscale (c), i.e., ER ratio = E/(R × c). Occupational stress is assumed to occur when the ER ratio is above 1, indicating an imbalance between efforts spent and rewards received in return (Siegrist et al. 2004). However, categorizing work stress using arbitrary cut off points for variables lacks natural or clinically based thresholds so far (van Vegchel et al. 2005).

### Statistical analysis

Frequencies, percentages (for categorical variables) as well as means and standard deviations (for continuous variables) were generated as applicable. Missing values in variables ranged between *n* = 3 for the subscale ‘cultural knowledge’ and *n* = 51 for ‘job experience’. Normal distribution of dependent variables for bivariate analyses was assessed graphically with the quantile–quantile (Q–Q) plot, since available analytical tests, e.g., the Shapiro–Wilk test, are highly sensitive with regard to sample size. Relationships between participants’ effort, reward, overcommitment and ER ratio and categorical variables were tested using independent t-tests or one-way ANOVA with post-hoc tests, respectively. Equivalently, we used Spearman correlation tests to test the latter relationships with continuous variables. As one precondition for multivariable analyses, normal distribution of residuals was plotted and assessed in histograms. Finally, multiple regression analyses were applied to determine variables associated with ER ratio, which was measured on a continuous scale as recommended by Montano et al. (2016) and van Vegchel et al. (2005). Supplementary analyses used effort, reward, and overcommitment as outcomes, respectively. Analyses were performed using IBM SPSS Statistics for Windows, version 25.0 (IBM Corp., Armonk, NY). *P*-values of < 0.05 were considered to indicate statistical significance.

## Results

Descriptive results of the study population are displayed in Table 1. The majority of participants was female (69.0%), had no migration experience (78.3%), was in the nursing profession (69.6%) and was employed in organization B (66.1%). Mean age was 41.3 years (standard deviation (SD): 11.1) and mean job experience was 16.6 years (SD: 12.0). Furthermore, the majority of participants did not hold a leading position (72.2%), held permanent contracts (73.6%) and worked full time (62.2%).

**Table 1:** Characteristics of participants and study settings (*N* = 800)

	*n*	%
Gender		
Female	536	69.0
Male	241	31.0
Age (years)		
Mean (SD)	41.3 (11.1)	
Job experience (years)		
Mean (SD)	16.6 (12.0)	
Migration experience		
No migration experience	610	78.3
Migration experience (first generation)	86	11.0
Migration experience (second generation)	83	10.7
Job role		
Physician	243	30.4
Nurse	557	69.6
Leading position		
Yes	221	27.8
No	573	72.2
Employment status		
Temporary	207	26.4
Permanent	578	73.6
Work status		
Full time	492	62.2
Part time	299	37.8
Experiences of discrimination		
Witness of discrimination (yes)	305	38.1
Victim of discrimination (yes)	108	13.5
Burden due to language barriers in interactions with patients^1^	3.3 (0.92)	
Burden due to language barriers in interactions with colleagues and supervisors^1^	2.2 (1.10)	
Cultural competence^1^		
Cultural knowledge (mean, SD)	3.5 (0.75)	
Cultural skills (mean, SD)	3.5 (0.60)	
Cultural metacognition (mean, SD)	3.5 (0.69)	
Total scale (mean, SD)	3.5 (0.55)	
Effort-reward imbalance		
Effort-reward ratio (mean, SD)	1.33 (0.42)	
Effort^2^ (mean, SD)	9.72 (1.75)	
Reward^3^ (mean, SD)	17.90 (3.36)	
Overcommitment^4^ (mean, SD)	14.73 (3.31)	
Institution		
A	271	33.9
B	529	66.1
Possibility to consult an interpreter in case of language barriers^1^	2.9 (1.06)	
Proportion of employees with a migration experience on ward in % (mean, SD)	22.1 (17.5)	
Proportion of patients with a migration experience on ward in % (mean, SD)	30.5 (20.5)	

^1^Scale range: 1–5; ^2^scale range: 3–12 points; ^3^scale range: 7–28 points; ^4^scale range: 6–24 points

Respondents estimated that a mean of 22.1% (SD: 17.5) of employees on their ward had a migration background, while a mean of 30.5% (SD: 20.5) of patients were assigned a migration background. Thirty-eight percent of participants reported witnessing an act of discrimination on their ward and 13.5% of respondents reported an own experience of discrimination. Participants described frequent burden due to language barriers in interactions with patients, but rather infrequent burden due to respective barriers in colleague and supervisor interactions. Respondents described average possibilities to consult interpreters in case of language barriers. Mean cultural competence was 3.5 (SD: 0.55) without considerable variance in the subscales of cultural knowledge, skills and metacognition.

The mean ER ratio was 1.33 (SD: 0.42), whereby mean effort was 9.72 (SD: 1.75), mean reward 17.90 (SD: 3.36) and mean overcommitment 14.73 (SD: 3.31). When ERI scores were dichotomized, 22.8% (*n* = 174) of respondents had ratios equal to or under 1 implying a favorable relationship between effort and reward spent at work. However, 77.2% (*n* = 590) of participants reported an imbalance between effort and reward.

Bivariate analyses revealed multiple associations between individual and organizational characteristics and the ER ratio, effort, reward, and overcommitment (see Tables 2, 3). A lower ER ratio was associated with a first generation migration background, being a physician, holding a leading position, having a temporary contract, working full-time, not having been a witness or victim of discrimination and reporting infrequent burden due to language barriers with patients and colleagues. On the other hand, higher ER ratios were associated with a lack of access to interpreter services in case of need and a higher proportion of patients with a migration background on one’s own ward. The above described variables were further associated with effort, reward or both. However, higher overcommitment was related to female gender, having been a witness or victim of discrimination, and frequent burden due to language barriers with patients and colleagues as well as missing interpreter access.

**Table 2:** Differences in effort-reward (ER) imbalance regarding individual and organizational characteristics

	ER ratio		Effort		Reward		Overcommitment	
	Mean	SD	Mean	SD	Mean	SD	Mean	SD
Gender^a^								
Female	1.34	0.42	9.75	1.74	17.83	3.27	14.93**	3.27
Male	1.31	0.42	9.63	1.74	18.10	3.54	14.19	3.29
Migration experience^b^								
No migration experience	1.35	0.42	9.84	1.71	17.85	3.37	14.69	3.27
Migration experience (first generation)	1.17***	0.36	8.89***	1.82	18.52	2.94	14.62	3.50
Migration experience (second generation)	1.30	0.41	9.67*	1.74	18.17	3.52	14.96	3.28
Job role^a^								
Physician	1.21***	0.37	9.36***	1.70	18.96***	3.49	14.78	3.23
Nurse	1.38	0.43	9.87	1.74	17.43	3.20	14.70	3.35
Leading position^a^								
Yes	1.25***	0.36	9.76	1.78	19.04***	3.36	14.70	3.43
No	1.37	0.44	9.70	1.73	17.46	3.27	14.73	3.28
Employment status^a^								
Temporary	1.25**	0.40	9.39***	1.84	18.43**	3.33	14.86	3.19
Permanent	1.36	0.42	9.85	1.69	17.69	3.35	14.69	3.36
Work status^a^								
Full time	1.30**	0.38	9.69	1.72	18.24***	3.33	14.83	3.34
Part time	1.39	0.48	9.76	1.80	17.31	3.34	14.53	3.26
Experiences of discrimination^a^								
Witness of discrimination								
Yes	1.43***	0.44	9.95**	1.70	17.06***	3.27	15.20**	3.38
No	1.27	0.39	9.57	1.76	18.42	3.32	14.43	3.24
Victim of discrimination								
Yes	1.49***	0.46	10.00	1.55	16.43***	3.22	15.55**	3.56
No	1.31	0.41	9.67	1.77	18.13	3.33	14.60	3.25
Institution^a^								
A	1.32	0.40	9.77	1.74	18.16	3.38	14.95	3.27
B	1.34	0.43	9.69	1.75	17.77	3.35	14.62	3.33

*SD* standard deviation. ^a^independent *t* test; ^b^one-way ANOVA with post-hoc Bonferroni tests. *p < 0.05, **p < 0.01, ***p ≤ 0.001

**Table 3:** Correlations between metrical study variables

		1	2	3	4	5	6	7	8	9	10	11	12	13	14	15
1	Age (years)	1														
2	Job experience (years)	0.87***	1													
	Effort-reward imbalance															
3	ER ratio	0.01	0.11**	1												
4	Effort	0.03	0.12***	0.79***	1											
5	Reward	− 0.00	− 0.07	− 0.81***	− 0.32***	1										
6	Overcommitment	0.01	0.03	0.41***	0.40***	− 0.25***	1									
	Cultural competence															
7	Cultural knowledge	− 0.10**	− 0.17***	− 0.01	− 0.07*	− 0.03	0.04	1								
8	Cultural skills	− 0.11**	− 0.16***	− 0.07*	− 0.07*	0.05	0.01	0.51***	1							
9	Cultural metacognition	− 0.04	− 0.06	− 0.05	− 0.04	0.04	0.00	0.43***	0.58***	1						
10	Total score	− 0.11**	− 0.17***	− 0.07	− 0.09*	0.04	0.02	0.73***	0.92***	0.76***	1					
11	Burden due to language barriers with patients	− 0.09*	− 0.08*	0.21***	0.21***	− 0.10**	0.11**	0.00	− 0.16***	− 0.05	− 0.12***	1				
12	Burden due to language barriers with colleagues and supervisors	0.11***	0.17***	0.19***	0.18***	− 0.14***	0.10**	− 0.10***	− 0.14***	− 0.08*	− 0.14***	0.28***	1			
13	Possibility to consult an interpreter	− 0.14***	− 0.17***	0.18***	0.09*	− 0.21***	0.12***	− 0.02	− 0.07*	− 0.08*	− 0.08*	0.06	0.00	1		
14	Proportion of employees with a migration experience on ward (%)	− 0.18***	− 0.19***	0.01	− 0.01	− 0.04	0.00	0.12***	0.11***	0.06	0.12***	− 0.02	− 0.06	− 0.00	1	
15	Proportion of patients with a migration experience on ward (%)	− 0.20***	− 0.14***	0.10**	0.10**	− 0.07	0.07	0.13***	0.13***	0.11**	0.15***	− 0.22***	0.02	− 0.06	0.43***	1

Spearman’s rho correlation coefficient. *p < 0.05, **p < 0.01, ***p ≤ 0.001

Multivariable analyses controlled for the simultaneous effects of individual and organizational characteristics on the ER ratio (see Table 4). Higher imbalance between effort and reward was associated with longer job experience (*β* = 0.092, *p* < 0.05), not holding a leading position (*β* = 0.122, *p* < 0.01), having been a witness (*β* = 0.149, *p* < 0.001) or victim of discrimination (*β* = 0.099, *p* < 0.05) and reporting frequent burden due to language barriers with patients (*β* = 0.102, *p* < 0.01) and colleagues (*β* = 0.127, *p* < 0.001). Higher ERI was further associated with lacking possibilities to consult interpreters in case of language barriers (*β* = 0.175, *p* < 0.001). However, lower ER ratios were related to a first generation migration background (*β* = − 0.095, *p* < 0.05) and being a physician (*β* = − 0.112, *p* < 0.05). In comparison to bivariate analyses, the associations of ERI with the employment status, work status and proportion of patients with a migration background on one’s ward were no longer statistically significant. This model explained 17.2% of the variance in the outcome ER ratio (adjusted *R*^2^ = 0.172). Additional calculations containing effort, reward, and overcommitment as dependent variables in multiple linear regression analyses are displayed in Online Resource Tables S1, S2, and S3.

**Table 4:** Multiple linear regression analyses of individual and organizational variables on effort-reward imbalance

	*B*	SE	*β*	95% CI (for B)
Constant	0.439	0.222		0.002; 0.876
Gender (female)	−0.025	0.034	− 0.028	− 0.091; 0.041
Job experience (in years)	**0.003**	**0.002**	0.092*	**0.000; 0.006**
Migration experience				
No migration experience	1	1	1	1
Migration experience (first generation)	**− 0.126**	**0.051**	− 0.095*	**− 0.227;− 0.026**
Migration experience (second generation)	**− **0.036		**− **0.026	−0.137; 0.060
Job role (physician)	**− 0.102**	**0.043**	− 0.112*	**− 0.186;− 0.017**
Leading position (no)	**0.114**	**0.040**	0.122**	**0.036; 0.192**
Employment status (permanent)	0.050	0.045	0.053	−0.038; 0.138
Work status (part time)	0.046	0.034	0.053	−0.022; 0.113
Experiences of discrimination				
Witness of discrimination (yes)	**0.127**	**0.033**	0.149***	**0.061; 0.193**
Victim of discrimination (yes)	**0.118**	**0.048**	0.099*	**0.024; 0.211**
Burden due to language barriers with patients	**0.048**	**0.019**	0.102**	**0.012; 0.085**
Burden due to language barriers with colleagues and supervisors	**0.049**	**0.015**	0.127***	**0.019; 0.078**
Cultural competence	**− **0.017	**− **	**− **	−0.074; 0.040
Institution (B)	**− **0.054	0.038	−0.062	−0.129; 0.020
Possibility to consult an interpreter	**0.069**	**0.015**	0.175***	**0.039; 0.098**
Proportion of employees with migration experience on ward (in %)	0.001	0.001	0.038	−0.001; 0.003
Proportion of patients with migration experience on ward (in %)	0.001	0.001	0.057	−0.001; 0.003
R^2^ (adjusted R^2^)	0.194 (0.172)			

Statistically significant association parameters are printed in bold. *N =* 650; *B* unstandardized coefficient, *SE* standard error*,* *β* standardized coefficient, *CI* confidence interval; *p < 0.05, ** *p* < 0.01, ***p ≤ 0.001

## Discussion

The overall prevalence of an unfavorable ratio of efforts spent and rewards received at work in our sample was high in comparison to studies of a representative sample of the German working population (Li et al. 2019) and other studies of healthcare workers (Nguyen Van et al. 2018). With regard to our study aim, we found that the experience of an imbalance between effort and reward at work was associated with multiple sociocultural diversity factors for healthcare personnel. Thus, having been a witness or victim of discrimination at one’s own ward, having reported frequent burden due to language barriers with patients and colleagues and the absence of interpreter services in case of translation needs were positively associated with higher ERI at work. Further factors, such as migration background, individual job experience, holding a leadership position and differences between physicians and nurses influenced participants’ reports of effort and reward at work. The following discussion centers around the impact of sociocultural diversity work factors on the perceived imbalance of effort and reward, but also takes into account their specific associations with effort, reward, and overcommitment as separate constructs.

### ERI and sociocultural diversity work factors

Our results reinforce the notion that sociocultural diversity factors are associated with work stress in healthcare personnel. With regard to the individual concern of employees, we observed that witnessing or experiencing discriminatory actions in one’s workplace due to specific individual traits was associated with work stress, i.e., an imbalance between effort and reward. Here, being a witness or victim were both significantly associated with the reward experienced at work, but not effort. These findings are supported by previous research on the reduced occupational well-being of physicians and nurses after experiencing discrimination at work (Boateng and Adams 2016; Hu et al. 2019; Shields and Price 2002). Interestingly, health professionals who migrated to Germany from another country reported lower levels of ERI and specifically effort spent at work, as opposed to previous literature suggesting higher perceptions of effort in migrated workers (Pasca and Wagner 2011). We assume that our participants with a personal migration experience might be less susceptible to effort spent at work due to lower expectations of their work environment in comparison to health professionals without a migration experience or the descendants of migrants. Since we controlled for other possible explanatory factors of this relationship in our statistical analyses, e.g., individuals’ job experience, gender, and job role, an effective discrepancy in job profiles of migrated and non-migrated healthcare professionals regarding required effort seems unlikely.

Difficulties in communicating with team members and patients due to lacking joint language skills, increased ERI in our study. Specifically, communication barriers with patients and colleagues were associated with increased effort in our sample, whereby only language barriers with colleagues and supervisors were associated with reward. In a study of French female hospital nurses, low levels of communication between nurses were associated with higher ERI and indirectly with depressive symptoms (Jolivet et al. 2010). However, the mere share of patients or colleagues with a migration experience did not influence work stress in our sample of nurses and physicians, which suggests that diverse workplaces per se were not perceived as stressful but rather resulting challenges in communication and lack of access to instruments to dissolve barriers.

In this context, the inability to contact interpreters in case of language barriers with patients showed significant associations with work stress. This factor was further associated with decreased levels of reported reward at work. The encounter of healthcare professionals and patients from diverse sociocultural backgrounds is a dynamic process requiring awareness and sensitivity from staff to patients’ background, previous experiences, needs, and expectations during their hospital stay. Fulfilling these requirements does not only involve cultural competence by staff but also time, flexibility and a shared common ground of communication which involves interpreters in case of different main languages of staff members and patients (Degrie et al. 2017). Individual cultural competence of healthcare professionals is central to high quality patient care (Betancourt et al. 2003). However, our study results reinforce the importance of organizational resources in the mitigation of employees’ work stress, since individual cultural competence did not explain variance in the experience of work stress in our sample, i.e., might not serve as a mitigating factor of psychosocially stressful work environments. If organizational resources are not available to healthcare staff, they might perceive a disproportionate amount of effort spent to deal with these work demands, as in the case of navigating language barriers with team members and patients. In the light of increasing sociocultural diversity among healthcare staff and patients, missing organizational resources for dealing with the additional effort required to handle the complexity of diverse patient needs and team work challenges might lead to increased work stress, impaired health of professionals and a respective decline in care quality (Klein et al. 2011; Loerbroks et al. 2016), in spite of a highly culturally competent work force.

Finally, our study focused on a transactional theory of work stress, i.e., health professionals’ cognitive and emotional appraisals of the interaction with their work environment, using the ERI model (Tabanelli et al. 2008). Interactional work stress models, such as the Job Demands-Control model (Karasek Jr 1979), might be complementary in our understanding of the association of diverse workplaces and employees’ view of the structural characteristics of their interaction with the work environment. Due to the explorative nature of our study and the lack of respective theoretical and empirical studies, we refrain from suggesting a specific work stress theory in the study of the effects of diverse workplaces on work stress. However, we deem the ERI model suitable for the study of our research questions, also due to its relevance to predict health-related outcomes in health professionals and other employees in high-stress occupations such as bus drivers (Useche et al. 2017) and humanitarian workers (Jachens and Houdmont 2019).

### ERI and occupational factors of health professionals

In accordance with our multivariable results on the profession of respondents, a Swedish study of early-career health professionals (Enberg et al. 2013) and a Greek study of health professionals during the COVID-19 pandemic (Tzenetidis et al. 2021) found out that nurses experience higher levels of ERI in comparison to physicians. If job experience is treated as a surrogate variable for age, supporting evidence for the findings of our study are available: A systematic review of ERI in physicians indicated that increasing age was associated with increasing reports of imbalance in multiple studies (Le Huu et al. 2022). A cross-sectional study of Chinese emergency nurses supported our findings that higher ERI was associated with longer work experience and exposure to verbal violence at work. However, contrary to our results, a leadership position was associated with higher ERI in nurses (Tian et al. 2021). Thus, specific subgroups of healthcare professionals, i.e., nurses and older employees, might be especially vulnerable to experience an imbalance of effort and reward at work.

## Limitations

First and foremost, we report results of a cross-sectional survey limiting inferences about directional associations between analyzed constructs. Thus, the experience of an imbalance of effort and reward at work, although unlikely, might affect healthcare professionals’ experience and rating of work burden due to discriminatory events, language barriers with patients or the availability of interpreters. Furthermore, personality variables were not surveyed in our study, limiting our focus on professional and sociodemographic features of health professionals. Previous research showed that higher negative emotionality, lower sociability and higher activity predicted higher levels of ERI (Hintsanen et al. 2011). Also, other organizational work factors, such as working hours and staffing levels might explain further variance in the experience of ERI (Beschoner et al. 2020; Jolivet et al. 2010). Further, with regard to the intercultural focus of our study, we would like to point out that certain terms or expressions used in the ERI questionnaire may be subject to cultural variation thus leading to differing understandings of respective items by respondents with a more limited command of the German language or another cultural background (Montano et al. 2016). Our survey data are based on self-report only, which makes it susceptible to systematic biases during data collection (Tabanelli et al. 2008). However, the main topics of this paper concern personal evaluations of experiences at work and may thus be best measured with self-report instruments (Montano et al. 2016). Online surveys often elicit low response rates as observed in our study. Sociodemographic information on the total population of contacted staff from the two participating organizations was not available for non-responder analysis. Only the distribution of professions across populations, i.e., the share of physicians and nurses in our sample and in the surveyed organizations, was comparable. Post hoc, a definitive response rate cannot be ascertained since we lack the total number of invited professionals who effectively read our email with the invitation to participate in the survey. Other studies with health professionals showed that men, persons aged 60 years and older as well as those working in specific national regions had higher non-response rates than women and younger persons. However, multiple survey reminders, as applied in our study, mitigated response bias due to differences in clinicians’ workload and their interest in the surveyed topic (Aerny-Perreten et al. 2015). Nonetheless, our study results have to be interpreted in light of the fact that the study sample might not be representative for the total population of invited professionals of the two organizations. Finally, attention must be drawn to the fact that differences in the prevalence of ER ratios were observed in a systematic review with regard to the response scale used to rate items, i.e., higher ERI prevalence in studies using four-point Likert scales, as applicable in our study, in comparison to studies applying five-point Likert response scales (Le Huu et al. 2022). In our study sample, 77.2% of respondents reported an imbalance between effort and reward, i.e., an ER ratio higher than 1, which is in line with the combined ER rate of studies using the four-point Likert scales for assessment in a systematic review of ERI in physicians (Le Huu et al. 2022). However, our study’s rate is much higher than most of the reported ER rates for health workers in the review by Nguyen Van et al. (2018).

## Conclusions

Hospital work environments are increasingly characterized by socioculturally diverse patient populations as well as care and medical teams. Furthermore, a considerable share of nurses and physicians perceive an imbalance between their effort spent at work and rewards received in return. Sociocultural diversity work factors, such as discrimination incidents at the workplace, language barriers with patients and colleagues, and unavailability of translation services are positively associated with higher work stress. However, the experience of imbalance further varies with regard to the profession, work experience, migration background and leadership position of health professionals under study. Organizational changes, such as the provision of adequate personnel and time resources might enable healthcare staff to deal with the increased challenges of communication and interaction within a socioculturally diverse work environment and might help to mitigate work stress and increase professionals’ job satisfaction as well as improve patient satisfaction with hospital care. Thus, potential interventions might address different levels to strengthen the cultural competence of healthcare systems (Betancourt et al. 2003): Organizational interventions might reinforce diversity-sensitive recruitment practices for leadership and health professionals to increase the share of minoritized persons in respective positions. Structural interventions might tackle the extension of hospital interpreter services and points of contact or trusted third parties for employees experiencing discrimination at work. Finally, individual provider level approaches might include cultural competence training to strengthen the individual skill set of healthcare professionals in the interaction with colleagues and patients from different sociocultural backgrounds. In the end, catering to the specific needs of healthcare personnel to deal with sociocultural diversity at the workplace and designing favorable work environments might be one cornerstone for the retention of qualified hospital staff and attraction of younger school graduates for a career in healthcare.

## Supplementary Information

Below is the link to the electronic supplementary material.

Supplementary file1 (PDF 192 KB)

Supplementary file2 (PDF 192 KB)

Supplementary file3 (PDF 192 KB)

## Data Availability

The datasets generated and analyzed during the current study are not publicly available due to data protection concerns, particularly on the part of the participating hospital organizations, but are available from the corresponding author on reasonable request.
