# Supplementary material for: Effort-reward imbalance and its association with sociocultural diversity factors at work: findings from a cross-sectional survey among physicians and nurses in Germany
Source: Int Arch Occup Environ Health. 2023 Jan 5;96(4):537–49. doi: 10.1007/s00420-022-01947-4 (PMC9812741; doi:10.1007/s00420-022-01947-4)
Supplement: Supplementary file 1 — Supplementary file1 (PDF 192 KB) [file 420_2022_1947_MOESM1_ESM.pdf]

Article title: Effort-reward imbalance and its association with sociocultural diversity factors at work: Findings from a cross-sectional survey among physicians and nurses in Germany

Journal name: International Archives of Occupational and Environmental Health

Author names: Anna Schneider, Christian Hering, Lisa Peppler, Liane Schenk

Affiliation and email-address: Institute of Medical Sociology and Rehabilitation Science, Charité – Universitätsmedizin Berlin, Berlin, Germany; anna.schneider@charite.de

Online Resource Table S1. Multiple linear regression analyses of individual and organizational variables on effort

	B	SE	β	95% CI (for B)
Constant	7.563	.934		5.730; 9.396
Gender (female)	-.036	.143	-.010	-.318; .245
Job experience (in years)	.010	.007	.072	-.002; .023
Migration experience				
No migration experience	1	1	1	1
Migration experience (first generation)	**-.680**	**.219**	**-.122****	**-1.110; -.249**
Migration experience (second generation)	-.129	.215	-.023	-.551; .293
Job role (physician)	-.238	.184	-.063	-.599; .122
Leading position (no)	-.011	.169	-.003	-.342; .321
Employment status (permanent)	.200	.190	.051	-.174; .573
Work status (part time)	.010	.146	.003	-.277; .296
Experiences of discrimination				
Witness of discrimination (yes)	.174	.142	.049	-.106; .453
Victim of discrimination (yes)	.210	.202	.042	-.187; .606
Burden due to language barriers with patients	**.361**	**.079**	**.186*****	**.206; .516**
Burden due to language barriers with colleagues and supervisors	**.136**	**.064**	**.085***	**.010; .263**
Cultural competence	-.076	.124	-.024	-.319; .168
Institution (B)	-.134	.161	-.037	-.450; .183
Possibility to consult an interpreter	.120	.064	.073	-.005; .245
Proportion of employees with migration experience on ward (in %)	.001	.004	.012	-.007; .009
Proportion of patients with migration experience on ward (in %)	.007	.004	.081	.000; .014
R^2 (adjusted R^2)	.126 (.103)			

Note: Significant association parameters are printed in bold; N = 671; B = unstandardized coefficient, SE = standard error, β = standardized coefficient, CI = confidence interval; * p < .05, ** p < .01, *** p ≤ .001.
