# Supplementary material for: Effort-reward imbalance and its association with sociocultural diversity factors at work: findings from a cross-sectional survey among physicians and nurses in Germany
Source: Int Arch Occup Environ Health. 2023 Jan 5;96(4):537–49. doi: 10.1007/s00420-022-01947-4 (PMC9812741; doi:10.1007/s00420-022-01947-4)
Supplement: Supplementary file 2 — Supplementary file2 (PDF 192 KB) [file 420_2022_1947_MOESM2_ESM.pdf]

Article title: Effort-reward imbalance and its association with sociocultural diversity factors at work: Findings from a cross-sectional survey among physicians and nurses in Germany

Journal name: International Archives of Occupational and Environmental Health

Author names: Anna Schneider, Christian Hering, Lisa Peppler, Liane Schenk

Affiliation and email-address: Institute of Medical Sociology and Rehabilitation Science, Charité – Universitätsmedizin Berlin, Berlin, Germany; anna.schneider@charite.de

Online Resource Table S2. Multiple linear regression analyses of individual and organizational variables on reward

	B	SE	B	95% CI (for B)
Constant	24.606	1.755		21.159; 28.053
Gender (female)	.283	.265	.040	-.238; .804
Job experience (in years)	**-.029**	**.012**	**-.104***	**-.053; -.005**
Migration experience				
No migration experience	1	1	1	1
Migration experience (first generation)	.292	.405	.027	-.503; 1.086
Migration experience (second generation)	.119	.410	.011	-.686; .925
Job role (physician)	**1.073**	**.337**	**.148****	**.411; 1.736**
Leading position (no)	**-1.501**	**.315**	**-.201*****	**-2.120; -.881**
Employment status (permanent)	-.288	.353	-.038	-.981; .406
Work status (part time)	-.332	.272	-.048	-.867; .202
Experiences of discrimination				
Witness of discrimination (yes)	**-1.109**	**.265**	**-.163*****	**-1.629; -.589**
Victim of discrimination (yes)	**-1.168**	**.378**	**-.122****	**-1.910; -.426**
Burden due to language barriers with patients	-.076	.147	-.020	-.365; .213
Burden due to language barriers with colleagues and supervisors	**-.352**	**.119**	**-.116****	**-.586; -.119**
Cultural competence	.185	.231	.031	-.268; .638
Institution (B)	.296	.301	.042	-.294; .886
Possibility to consult an interpreter	**-.529**	**.118**	**-.168*****	**-.761; -.297**
Proportion of employees with migration experience on ward (in %)	-.004	.008	-.021	-.019; .011
Proportion of patients with migration experience on ward (in %)	-.007	.007	-.045	-.021; .006
R^2 (adjusted R^2)	.198 (.177)			

Note: Significant association parameters are printed in bold; N = 658; B = unstandardized coefficient, SE = standard error, β = standardized coefficient, CI = confidence interval; * p < .05, ** p < .01, *** p ≤ .001.
