# Supplementary material for: Effort-reward imbalance and its association with sociocultural diversity factors at work: findings from a cross-sectional survey among physicians and nurses in Germany
Source: Int Arch Occup Environ Health. 2023 Jan 5;96(4):537–49. doi: 10.1007/s00420-022-01947-4 (PMC9812741; doi:10.1007/s00420-022-01947-4)
Supplement: Supplementary file 3 — Supplementary file3 (PDF 192 KB) [file 420_2022_1947_MOESM3_ESM.pdf]

Article title: Effort-reward imbalance and its association with sociocultural diversity factors at work: Findings from a cross-sectional survey among physicians and nurses in Germany

Journal name: International Archives of Occupational and Environmental Health

Author names: Anna Schneider, Christian Hering, Lisa Peppler, Liane Schenk

Affiliation and email-address: Institute of Medical Sociology and Rehabilitation Science, Charité – Universitätsmedizin Berlin, Berlin, Germany; anna.schneider@charite.de

Online Resource Table S3. Multiple linear regression analyses of individual and organizational variables on overcommitment

	B	SE	B	95% CI (for B)
Constant	10.526	1.820		6.952; 14.100
Gender (female)	**.784**	**.281**	**.111****	**.232; 1.336**
Job experience (in years)	.014	.013	.049	-.012; .039
Migration experience				
No migration experience	1	1	1	1
Migration experience (first generation)	-.188	.431	-.018	-1.034; .658
Migration experience (second generation)	.053	.422	.005	-.776; .882
Job role (physician)	.139	.357	.019	-.562; .839
Leading position (no)	-.346	.336	-.046	-1.006; .314
Employment status (permanent)	-.247	.370	-.033	-.974; .481
Work status (part time)	-.412	.289	-.060	-.979; .155
Experiences of discrimination				
Witness of discrimination (yes)	.505	.280	.075	-.044; 1.055
Victim of discrimination (yes)	.589	.396	.063	-.187; 1.366
Burden due to language barriers with patients	.198	.155	.053	-.106; .503
Burden due to language barriers with colleagues and supervisors	**.322**	**.127**	**.106***	**.073; .571**
Cultural competence	.081	.244	.014	-.398; .560
Institution (B)	-.242	.318	-.035	-.866; .383
Possibility to consult an interpreter	**.450**	**.125**	**.145*****	**.206; .695**
Proportion of employees with migration experience on ward (in %)	-.001	.008	-.004	-.017; .016
Proportion of patients with migration experience on ward (in %)	.004	.007	.024	-.011; .018
R^2 (adjusted R^2)	.072 (.048)			

Note: Significant association parameters are printed in bold; N = 669; B = unstandardized coefficient, SE = standard error, β = standardized coefficient, CI = confidence interval; * p < .05, ** p < .01, *** p ≤ .001
